# Arc Discharges of a Pure Carbon Strip Affected by Dynamic Contact Force during Current-Carrying Sliding

**DOI:** 10.3390/ma11050796

**Published:** 2018-05-15

**Authors:** Yanyan Zhang, Yongzhen Zhang, Chenfei Song

**Affiliations:** 1 School of Mechanical Engineering, Northwestern Polytechnical University, Xi’an 710072, China; zhangyanyan21168@163.com; 2 National United Engineering Laboratory for Advanced Bearing Tribology, Henan University of Science and Technology, Luoyang 471023, China

**Keywords:** arc discharge, current carrying, pure carbon strip, dynamic contact force

## Abstract

Arc discharges of a pure carbon strip induced by dynamic contact force were studied on a pin-on-disk tribometer. It was found that arc discharges were produced periodically in accordance with the period of the dynamic contact force. The arcing rate of the pure carbon strip increased with an increase of frequency *f* and amplitude *B*, which led to a decrease of current-carrying quality. These influences at high velocities became much more significant. A critical point of the arcing rate at around 2% was detected. Lower than 2%, the pure carbon strip was able to maintain its excellent current-carrying capability; higher than this point, the current-carrying quality deteriorated abruptly. SEM and XPS analysis show that the element Cu detected on the worn surface at lower arcing rates was metal Cu. CuO was found at higher arcing rates. This indicated that the wear mechanism transferred from mechanical wear to arc erosion with the increase of the arcing rate.

## 1. Introduction

A pure carbon strip is one of the most important electric components on a pantograph for energy transformation from the railway electric distribution network to the train. Arc discharge is a common and typical phenomenon when the strip rubs against the contact wire. In order to study the damage mechanisms of arc discharges on electrical contact material, a large number of efforts have been devoted to research in this field in past decades [1,2,3,4]. For instance, Kubo et al. [5] studied the effects of arc discharge on the wear rate of a copper-impregnated metalized carbon strip at different numbers of revolutions and found that the accumulated discharge energy was proportional to the wear rate. Chen et al. [6] carried out a series of tests on arc discharge at different normal force, drawing the conclusion that arc discharge could be suppressed and the wear rate of the pure carbon strip could be decreased by increasing the normal force. Xiong et al. [7] investigated the wear behavior of a pure carbon strip under different electric current densities. Results showed that the degree of arc erosion increased with the increase of the electric current densities. Furthermore, Yang et al. [8] captured the dynamic variation of arc discharge using a high-speed camera. Observation results revealed that arc discharges were able to migrate along the sliding direction. In general, works presented in these investigations usually focused on arc discharge induced by static load [9,10,11,12,13]. It should be noted that the pantograph and catenary are typical dynamic contact friction pairs whose contact force has different frequencies and amplitudes at different velocities. The faster the train runs, the more influences the dynamic contact force has on the pantograph/catenary properties. However, the electrical performance subjected to dynamic contact force is still unclear. Therefore, it is necessary to study the arcing behaviors and their effects on pantograph/catenary current-carrying properties affected by the dynamic contact force.

This paper studied the arc discharge characteristics of a pure carbon strip influenced by dynamic contact force. The dynamic contact force in the pantograph/catenary system was calculated using the finite element method (FEM) [14,15,16,17,18], which showed that the frequency and amplitude of the dynamic contact force increased with the increase of running speed of the locomotives. Then, the dynamic contact force was sent to a pin-on-disk tribometer in the form of a sine wave for the tribological tests. The effects of frequency and amplitude on arcing rate were studied firstly. Then, the influence of the arcing rate on current-carrying quality and their damage to the pure carbon strip were studied. In particular, the worn surface of the strip was observed using a 3D surface profiler explorer. Arc erosion characteristics of the pure carbon strip were analyzed using a scanning electron microscope (SEM) and energy-dispersive X-ray spectroscopy (EDX). At last, the ablation products and wear debris produced during the current-carrying sliding were detected using XPS. This study obtained the arc discharge properties between the pure carbon strip and contact wire, with the expectation of providing useful suggestions for the safe operation of locomotives.

## 2. Materials and Methods

### 2.1. Test Materials

**Figure 1:**
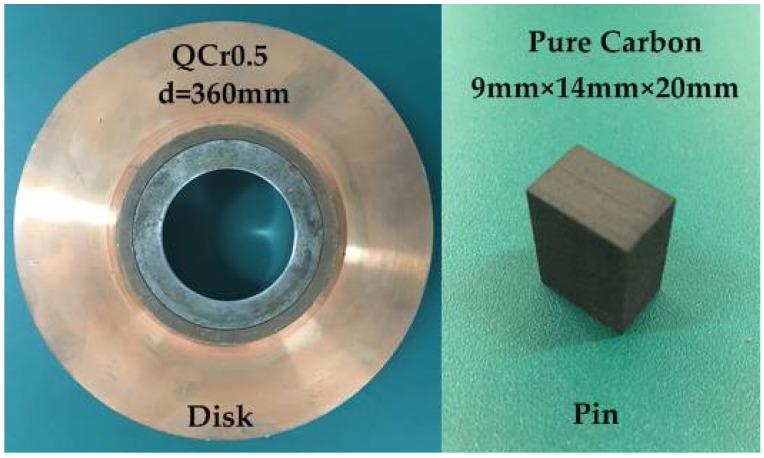
The QCr0.5 disk and the pure carbon pin.

A pure carbon strip and QCr0.5 (GB/T 13808-1992) were chosen as the tested friction couples. The pure carbon strips are currently in service for the China railway. They are used to transmit electricity from the electric distribution network to the trains by sliding along the contact wire. The pure carbon strip employed in the experiments came from the DSA200 pantograph on an SS7 electric locomotive (CRRC Datong Co. Ltd., China) provided by Luoyang Locomotive Depot. It was made up of 0.95 C and 0.05 S, N, Cl, etc. The pure carbon strips were cut into pins of 9 mm × 14 mm × 20 mm for tribological tests. QCr0.5 is the contact wire material which composed of Cu, 0.5 Cr, 0.005 Ni, 0.005 Fe, 0.005 Zn, 0.005 Pb, etc. It was made into a disk specimen whose diameter was 180 mm, as seen in Figure 1. The relative movement between the pantograph and the contact wire was simulated by the pin rubbing against the rolling disk. Prior to each electrical sliding test, the pin samples were preworn on the test rig under 50 N and 10 m·s^−1^ for 60 s without electricity.

The physical properties of the pure carbon strip are exhibited in Table 1. Table 2 shows the chemical composition and mass fraction of QCr0.5.

### 2.2. Test Equipment

The pantograph/catenary system was composed of two separated elastic systems, which were coupled together by the dynamic contact force. In order to achieve the dynamic contact force for the following tribological tests, a Finite Element Method (FEM) model of the pantograph/catenary system was founded referencing the DSA200 pantograph. They are currently in service in China on the SS7 electric locomotive [19,20,21]. The pantograph is installed on top of the locomotive and slides along the contact wire with the traveling of the train. It was modeled as a mass-spring-damper system with three degrees of freedom. The catenary system is composed of contact wire, messenger wire, droppers, bracket, registration arm, etc. It was modeled as a simple catenary system. For preliminary experiments, the FEM model did not take account of the track vibration and aerodynamic force. FEM results showed that the frequency and amplitude of the dynamic contact force increased with the increase of the running speed. The dynamic contact force fluctuated periodically with each span [22,23,24,25,26,27], which can be described by the fundamental wave as Equation (1):

(1) F(t)=70+Bsin(2πft)

where *B* is the amplitude of the dynamic contact force and *f* is the frequency. The *f* and *B* of the dynamic contact force at different velocities were calculated by Fast Fourier Transform (FFT), shown in Table 3. Finally, these parameters were sent to a homemade pin-on-disk tribometer (HST-100, China) with current at 120 A. Arc discharges of the pure carbon strip subjected to dynamic contact force at different velocities were captured on the test rig.

**Figure 2:**
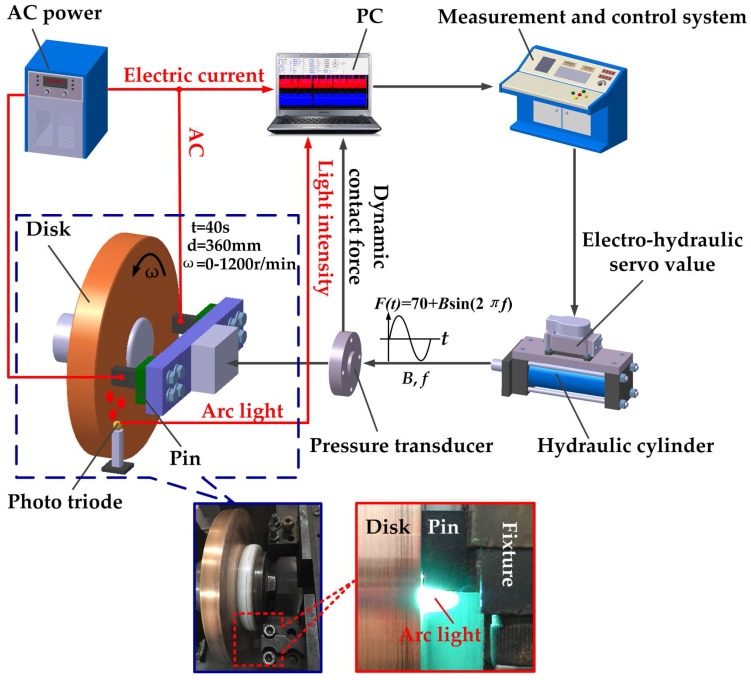
Schematic diagram of the test rig.

A schematic diagram of the HST-100 pin-on-disk tribometer is shown in Figure 2. The electrohydraulic servo value was used to switch the dynamic contact force into the corresponding pressure signal. The dynamic contact force can be loaded in form of a sine wave, triangular wave, or square wave with different amplitudes and frequencies. The loading system is able to endure an impacting amplitude of 500 N with a maximum frequency of 50 Hz. The input parameters are continuously adjustable. As a result, the test rig was capable of simulating the flexible contact in a pantograph/catenary system. A pressure transducer was employed to record the real dynamic contact force loaded on the pins. Moreover, AC power supplied electricity to the pins up to 300 A. Arc light during the tests was captured using a photo triode. Finally, electric current, light intensity, and dynamic contact force were sent to a PC for further study after wear tests. A scanning electron microscope (JSM-5610LV, JEOL, Tokyo, Japan) was employed to observe the worn surface. A 3D surface profiler explorer (NanoFocus-AG, Oberhausen, Germany) was used to observe the surface profile and the surface roughness profile. The chemical properties of the worn surfaces were detected using XPS (Axis Supra, Kratos, Manchester, UK).

The linear sliding speed between the disk and the pins was varied from 0 to 100 m/s (12,000 r/min) to simulate the relative movement of the pantograph and the contact wire. All the current-carrying experiments were carried out with current at 120 A for 40 s. There were two different experiment methods in this study. At first, coupling experiments were conducted with the dynamic contact force parameters in Table 3. Then, decoupling experiments were designed for deeper study as discussed in the following section.

## 3. Results and Discussion

### 3.1. Arc Discharge Characteristics

**Figure 3:**
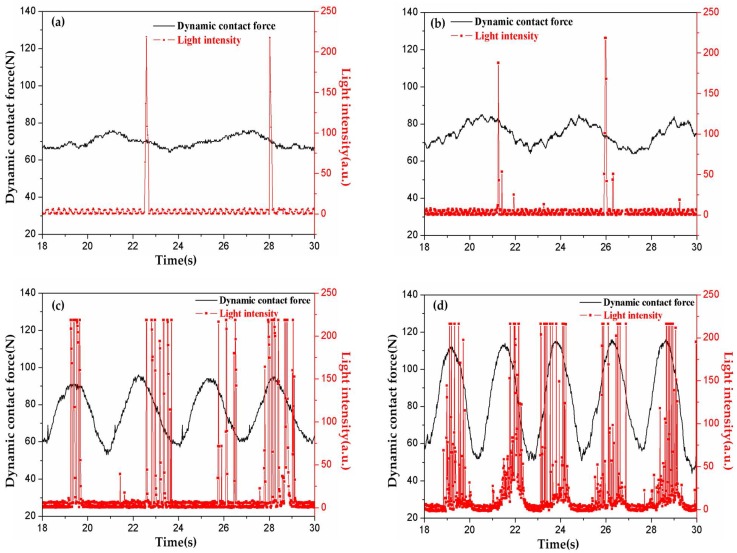
Light intensity varying with velocity: (**a**) 40 km·h^−1^; (**b**) 60 km·h^−1^; (**c**) 80 km·h^−1^; (**d**) 100 km·h^−1^.

**Figure 4:**
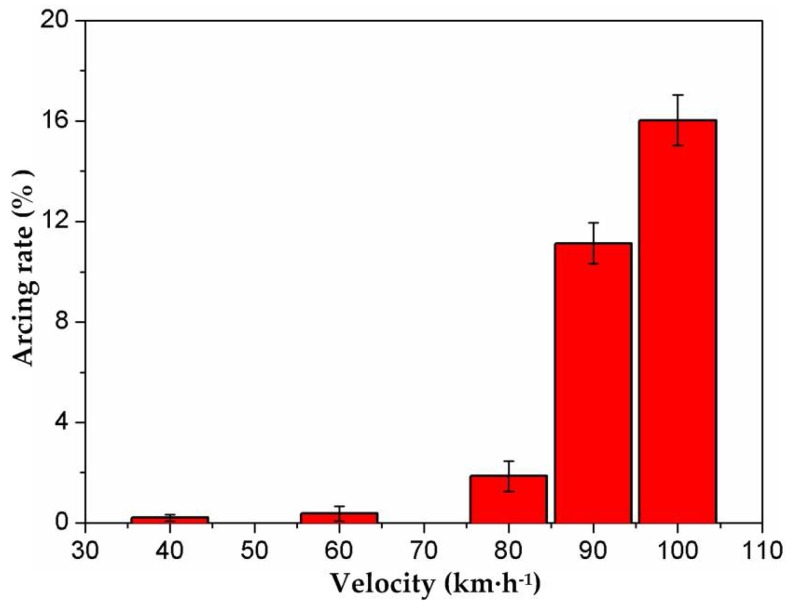
Arcing rate of the pure carbon strip varying with velocity.

Coupling experiments were carried out at different velocities with the dynamic contact force in Table 3. Arc discharges produced in the tests were captured using a photo triode, as shown in Figure 3. It was found that arc discharges were produced periodically in accordance with the period of the dynamic contact force. This is quite different from previous static studies, in which arc discharges were always produced randomly under constant load [6,8]. In addition, the number of arc discharges increased with the increase of the running speed within the same test duration. The arcing rate exhibited a rising tendency from 40 km·h^−1^ to 100 km·h^−1^, seen in Figure 4. What is special is that it raised abruptly to about 16% after 80 km·h^−1^, before which it was lower than 2% and raised slowly.

Still, it should be noted that the frequency (*f*) and amplitude (*B*) of the dynamic contact force increased simultaneously with the increase in velocity. In order to make clear the single effects of *f* and *B* on arc discharge, decoupling experiments were designed and carried out with current at 120 A. Three typical velocities were chosen according to the arcing rate in Figure 4, which were 40 km·h^−^^1^ at the low arcing rate, 100 km·h^−^^1^ at the high arcing rate, and the critical point of arcing rate at 80 km·h^−^^1^. The dynamic contact force shown in Table 4 was sent to the tribometer to study the effect of *f* and *B* on the arcing rate. As Equation (1) expressed, the base value of the dynamic contact force in this study was set at 70 N. Firstly, the experiments were carried out according to Test 1 at 40 km/h with *B* fixed at 8 N. Decoupling tests were conducted one by one from 0.169 Hz to 0.426 Hz to study the effect of *f.* Then, the tribological tests proceeded at 80 km/h with *B* fixed at 28 N, varying *f* from 0.169 Hz to 0.426 Hz. Thirdly, a running speed at 100 km/h with *B* at 41 N was tested with a series of *f* in Test 1. With the same methods, Test 2 was carried out at 40 km/h, 80 km/h, and 100 km/h to study the effect of *B*.

**Figure 5:**
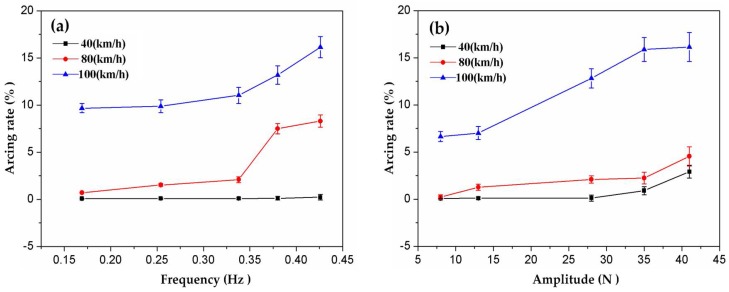
Arcing rate of the pure carbon strip varying with (**a**) frequency and (**b**) amplitude.

Affected by the dynamic contact force, the worn surfaces of the friction couples suffered the repeated impact of loading and unloading. The number of times of impact depended on *f*, while *B* determined the impacting strength. Figure 5 shows the decoupling experiment result which shows the individual effects of *f* and *B*. At 40 km·h^−1^, *f* had little influence on the arcing rate. It was maintained lower than 0.3%, shown in Figure 5a. At 80 km·h^−1^, the influence of *f* on the arcing rate was enhanced from 0.7% to 8.3%. When the train ran at 100 km·h^−1^, the arcing rate of the strip reached more than 10 times that produced at 40 km·h^−1^, which rose rapidly from 9.7% to 16.1% with increasing *f*. Similarly, the effect of *B* on the arcing rate at 100 km·h^−1^ was much stronger than those produced at 40 km·h^−1^ and 80 km·h^−1^, as seen in Figure 5b. In short, the increase of *f* and *B* led to the increase of the arcing rate. The degrees of influence were different at various velocities. The faster the train ran, the more influence the action of *f* and *B* had.

### 3.2. Effects of Arcing Rate on Current-Carrying Quality

**Figure 6:**
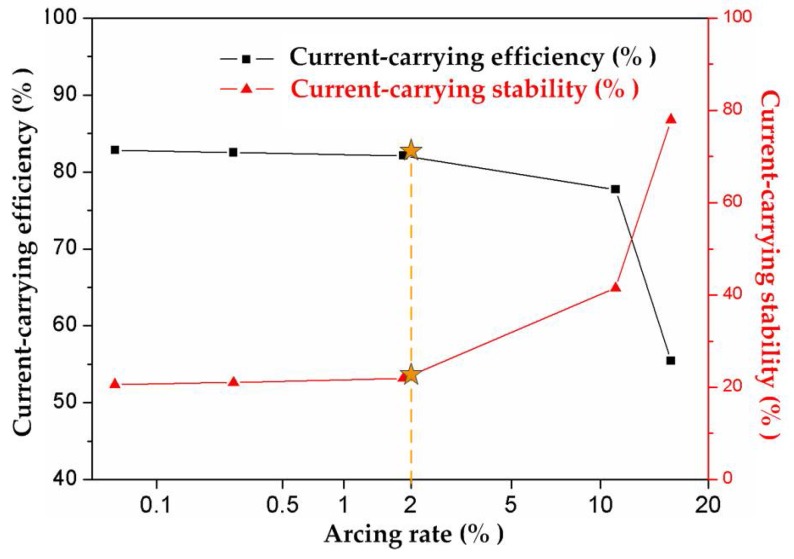
Current-carrying quality affected by arcing rate with the test parameters in Table 3.

Arc discharges produced during the current-carrying sliding resulted in the loss of electrical energy and the fluctuation of the current supplied to the trains. In order to evaluate the capabilities of energy transformation and the distortion characteristic of the pulse current between the strip and the contact wire, current-carrying quality is employed with two evaluation parameters: current-carrying efficiency and current-carrying stability. Current-carrying efficiency is defined as

(2) $$\eta =\frac{\bar{I}}{I_{0}}\times 100\%$$

where $\bar{I}$ is the average value of electrical current for the duration of the test and $I_{0}$ is the set value (120 A in this test). Current-carrying stability is defined as

(3) $$\delta =\frac{\sqrt{\frac{1}{n}\sum_{i=1}^{n}{\left(I_{i}-\bar{I}\right)}^{2}}}{\bar{I}}\times 100\%$$

where $I_{i}$ is the instantaneous current value. Both η and δ are dimensionless. Figure 6 shows the current-carrying efficiency and current-carrying stability varying with arcing rate produced by the coupling experiments in Table 3. With the increase of the arcing rate, the current-carrying efficiency of the strip was decreased. Accordingly, the current-carrying stability became worse. When the arcing rate was lower than 2%, it had no evident influence on the current-carrying quality. The current-carrying efficiency of the strip was maintained at 82% and the current-carrying stability stayed at about 20%. In particular, when the arcing rate exceeded 2%, the current-carrying quality declined abruptly. η was decreased to 53%, which signified the sharp degradation of the energy efficiency, and δ was lifted to 78%, suggesting the deterioration of the electrical current volatility.

**Figure 7:**
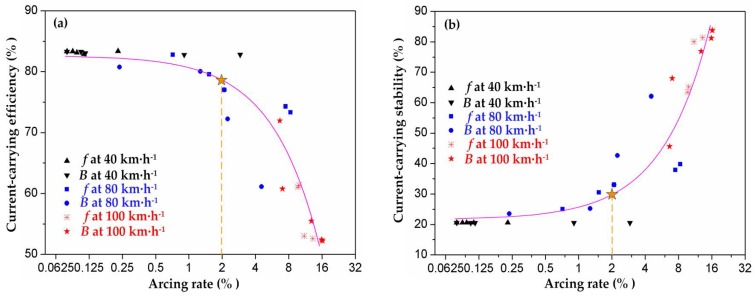
(**a**) Current-carrying efficiency and (**b**) current-carrying stability in decoupling experiments with the test parameters in Table 4.

Moreover, the current-carrying quality was calculated after the decoupling experiments in Table 4, as shown in Figure 7. It was detected that the increase of *f* and *B* led to the raising of the arcing rate, which in turn exhibited a negative impact on η and δ. Both *f* and *B* had little influence on the current-carrying quality at 40 km·h^−^^1^. η and δ were maintained at about 83% and 20.5%, respectively. The pure carbon strip showed good capacity for energy transmission at this time. Faster than 80 km·h^−^^1^, *f* and *B* began to exhibit damage to the current-carrying quality. At 100 km·h^−^^1^, η was reduced to 52% and δ was lifted to 83%, which disturbed the energy transmission dramatically.

Very interesting was the arcing rate at 2% which was found to be a critical point for the current-carrying quality, as seen in Figure 6 and Figure 7. The pure carbon strip was capable of maintaining its excellent energy transmission capability with arcing rate lower than 2%. However, when the arcing rate of the strip was higher than 2%, the current-carrying quality deteriorated abruptly.

### 3.3. Effects of Arc Discharge on Damage to the Pure Carbon Strip

**Figure 8:**
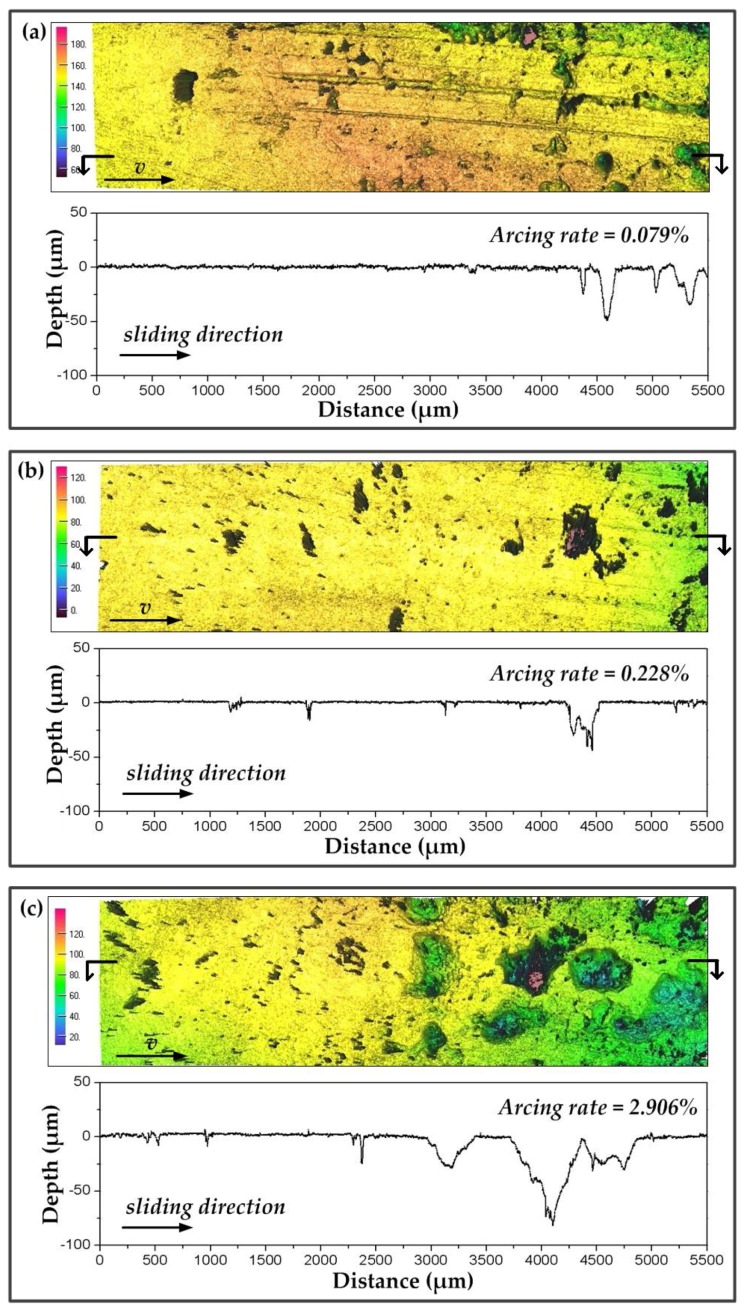
3D surface morphology (**top**) and surface roughness profiles (**bottom**) of the strip after testing for 40 s at 40 km·h^−^^1^, 120 A: (**a**) *B* = 8 N, *f* = 0.169 H; (**b**) *B* = 8 N, *f* = 0.426 Hz; (**c**) *B* = 41 N, *f* = 0.169 Hz.

**Figure 9:**
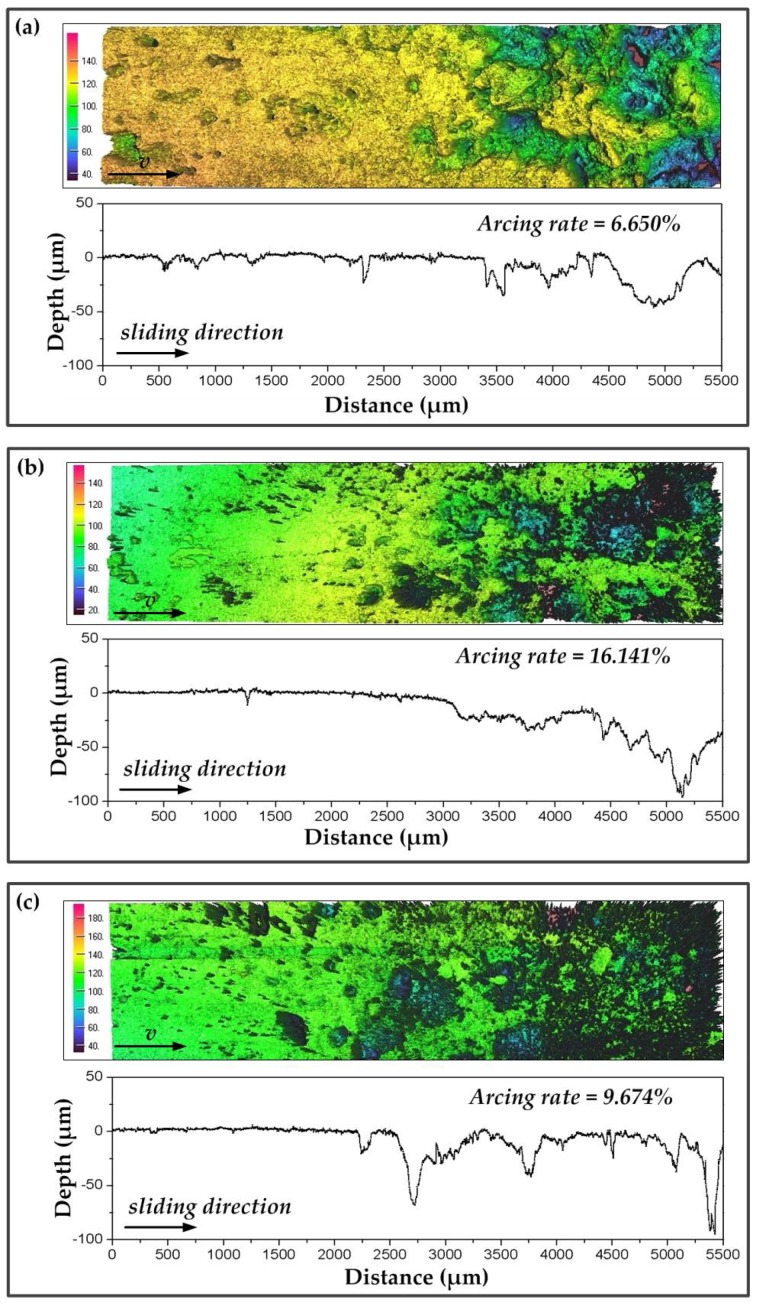
3D surface morphology (**top**) and surface roughness profiles (**bottom**) of the strip after testing for 40 s at 100 km·h^−^^1^, 120 A: (**a**) *B* = 8 N; *f* = 0.426 Hz; (**b**) *B* = 41 N, *f* = 0.426 Hz; (**c**) *B* = 41 N, *f* = 0.169 Hz.

The increasing *f* and *B* of the dynamic contact force caused the increase in arc discharge, by which the worn surface of the pure carbon strip was damaged. In this section, morphologies of the worn surfaces at different arcing rates were observed using a 3D surface profiler explorer. Strips at 40 km·h^−^^1^ (low arcing rate) and 100 km·h^−^^1^ (high arcing rate) were detected, and are shown in Figure 8 and Figure 9, respectively. On the whole, arc erosion pits always gathered at the end of the worn surface. The surface quality became poorer along the sliding direction. A previous study had found that arc discharges in current-carrying sliding exhibited a migrating characteristic under static load [8]. The migrating characteristic was also detected in this dynamic study. In addition, the worn surfaces of the strips at different *f* and *B* are discussed.

Scratches and little arc erosion pits were found in Figure 8a,b. The arcing rate of the strip was maintained at lower than 0.3%. The corresponding surface roughness profiles showed that the erosion depth was about 50 µm. Although *f* in Figure 8b was increased to 0.426 Hz, the erosion depth was similar to that in Figure 8a. However, when *B* was increased to 41 N with other parameters unchanged, the arcing rate of the strip was increased to 2.906%. Erosion pits in Figure 8c were found to be much larger and deeper. The surface roughness profiles showed that the erosion depth had reached about 80 µm, as shown in Figure 8f.

When the train speed was 100 km·h^−^^1^, the migrating characteristic of the arc discharges was even easier to detect, which can be found in Figure 9. Strong erosion morphology was observed at this high arcing level. Surface roughness profiles exhibited that the erosion depth increased along the sliding direction, which indicated the gradually aggravated arc erosion. Figure 9a shows the worn surface at 8 N, while Figure 9b shows the worn surface at 41 N. The arcing rate of the strip was increased from 6.65% to 16.141%. Dense and sharp peak-shaped asperities occupied most of the erosion area suffered from high amplitude, as seen in Figure 9b, which was much rougher than the worn surface in Figure 9a. The corresponding depth of the erosion pits in Figure 9b was increased to 100 µm. When *f* was decreased to 0.169 Hz in Figure 9c, the arcing rate of the strip was reduced to 9.674%. However, the surface quality of the pure carbon strip had no sufficient distinction from that in Figure 9b.

In general, the increase of amplitude from 8 N to 41 N induced the raising of the arcing rate and deterioration of the surface quality which left a number of arc erosion pits on the worn surface. However, the increase of frequency from 0.169 Hz to 0.426 Hz did not produce significant changes in the 3D surface morphologies. To have a better understanding of the arc erosion mechanism brought by amplitude, SEM and EDX of the worn surface at low arcing rates and high arcing rates were carried out. Furthermore, the oxidation products on the worn surfaces were detected by XPS.

### 3.4. Arc Erosion Characteristics

**Figure 10:**
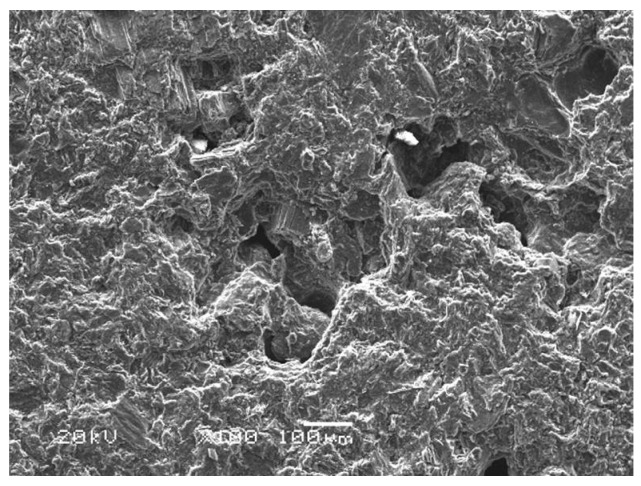
SEM morphology of the original surface.

**Figure 11:**
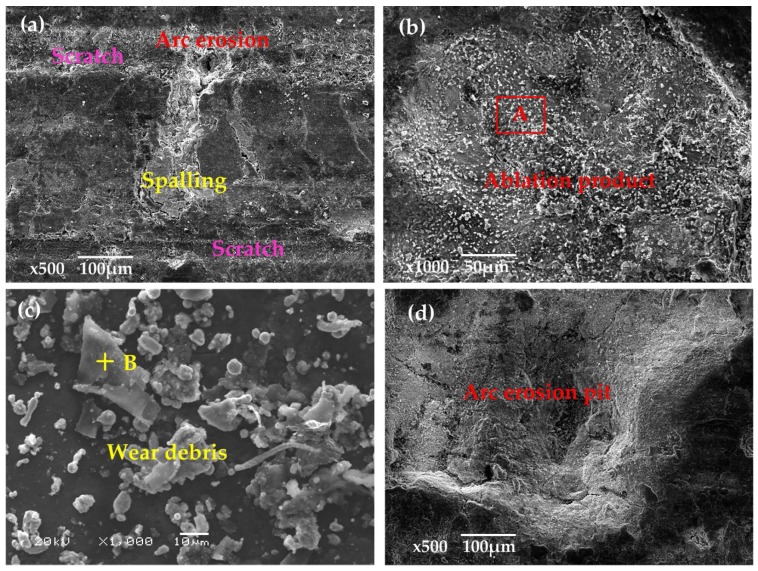
SEM of the worn surfaces at low arcing rate (40 km·h^−1^, 40 s, 120 A): (**a**) B = 8 N, *f* = 0.169 Hz, arcing rate = 0.079%; (**b**) ablation products from (**a**); (**c**) debris from (**a**); (**d**) B = 41 N, *f* = 0.169 Hz, arcing rate = 2.9%.

Figure 10 is the SEM image of the original surface of the pure carbon strip before testing. It was found that the strip was characterized by loose structure and irregular pore spaces. At 40 km·h^−^^1^, the arcing rate of the pure carbon strip was maintained at a low level (0.079%). Obvious scratches with small erosion pits were visible along the sliding direction in Figure 11a. The ablation products in the erosion pits were snowflake-shaped, with diameter of only 5 µm, which can be seen in Figure 11b. Comparatively, the wear debris outside the erosion pits were much larger. The largest diameter of these scale-like particles in the debris had reached to about 30 µm, presented in Figure 11c.

**Figure 12:**
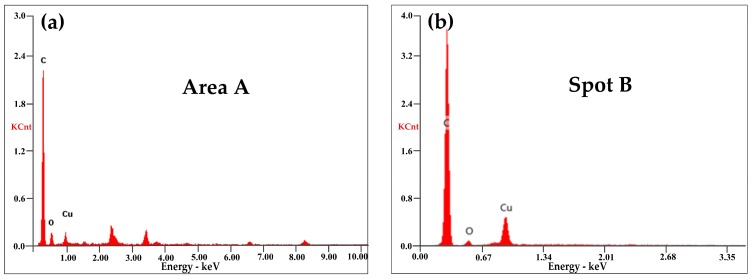
EDX of the selected area: (**a**) ablation products from Figure 11b; (**b**) debris from Figure 11c.

EDX in Figure 12a,b exhibited that both the ablation products and the wear debris in Figure 11 contained large amounts of C with only a little of Cu and O. The large chunks of carbon debris showed that mechanical wear was the main wear mechanism at a low arcing rate. Elemental Cu was transferred from the contact wire (QCr0.5) by abrasive wear and slight arc erosion. 

When *B* was increased to 41 N with other parameters unchanged, the arcing rate of the strip was increased to 2.9%, as shown in Figure 5b. Large erosion pits were produced, as seen in Figure 11d, which coincided with the 3D surface morphology in Figure 8c. The contact force loaded to the pure carbon strip at this working condition varied from 29 to 111 N (70 ± 41 N). The repeated impact of loading and unloading decreased the stability between the friction couples. When the gap voltage between the friction couples was greater than the minimum breakdown voltage, arc discharges took place [1,4]. 

**Figure 13:**
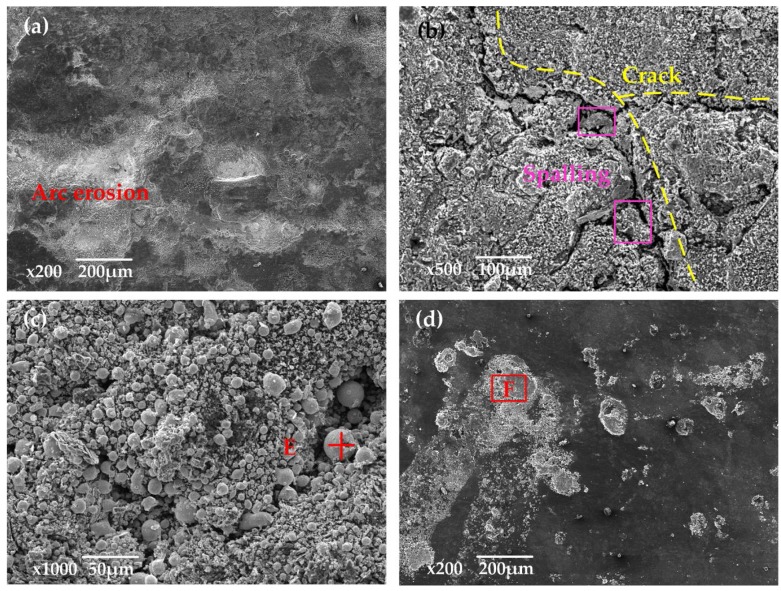
SEM of the worn surfaces at high arcing rate (100 km·h^−1^, 40 s, 120 A): (**a**) B = 8 N, *f* = 0.426 Hz, arcing rate = 6.65%; (**b**) B = 41 N, *f* = 0.426 Hz, arcing rate = 16.141%; (**c**) ablation products from (**b**); (**d**) debris from (**b**).

**Figure 14:**
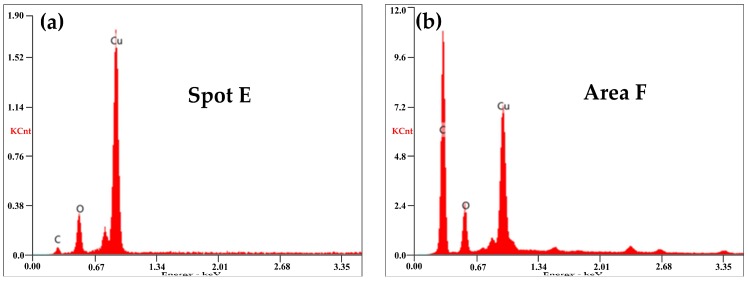
EDX of the selected area: (**a**) ablation products from Figure 13c; (**b**) debris from Figure 13d.

At 100 km·h^−1^, large erosion pits were produced on the worn surface, as seen in Figure 13a. When *B* was increased to 41 N, the arcing rate of the strip was suddenly increased to 16.141%. Suffered from intensive arc erosion with high frequency, the worn surface of the strip in Figure 13b was destroyed dramatically. Fierce arc light was visible and pungent smells mixed with white fumes were detected during the current-carrying tests. At the same time, the generation of cracks accompanied by the spalling of fragments accelerated the consumption of the pure carbon strip. The SEM in Figure 13c found a number of spherical particles around the cracks. The EDX in Figure 14a indicated that these spherical particles contained a lot of Cu with a small amount of C and O.

Comparing the EDX analysis in Figure 12 and Figure 14, it was found that the content of element Cu and O was much higher at 100 km·h^−^^1^. The wear and oxidation of the pure carbon strip at a high arcing rate was much more severe than that at a low arcing rate. This means that the damage mechanism changed at different arcing rates. To have better understanding of this, XPS was carried out on the worn surfaces in Figure 11a and Figure 13c which were produced by low arcing rate (0.079%) and high arcing rate (16.141%), respectively. The chemical state of Cu was focused on because Cu was oxidized and left a solid residue on the worn surfaces.

**Figure 15:**
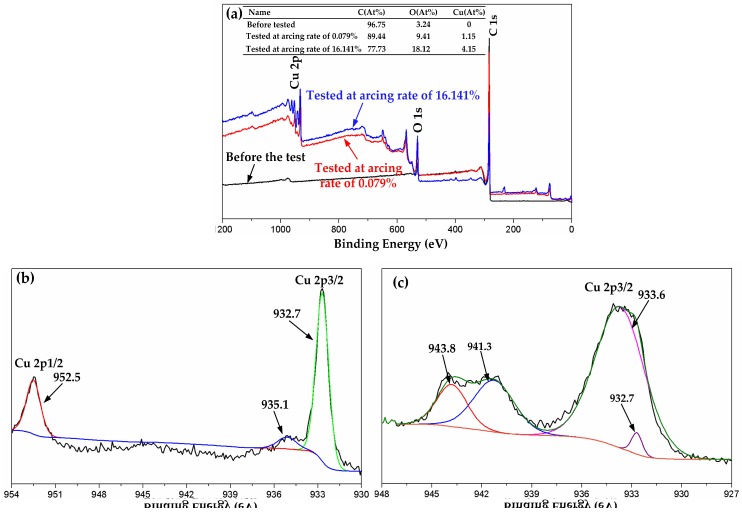
XPS test results: (**a**) XPS spectra of C 1s, O 1s and Cu 2p before and after the test at 120 A; (**b**) XPS spectra of Cu 2p at arcing rate of 0.079%; (**c**) XPS spectra of Cu 2p3/2 at arcing rate of 16.141%. Binding Energy was calibrated with C1s = 284.5 eV.

Figure 15a shows the XPS spectra of C 1s, O 1s, and Cu 2p of the ablation products before and after testing. Before testing, there was no elemental Cu in the pure carbon strip. Then, elemental Cu and an increase of O were detected in the ablation products after current-carrying sliding. XPS spectra of the worn surface in Figure 11a at a low arcing rate showed two well-formed Cu 2p peaks corresponding to the Cu 2p1/2 and Cu 2p3/2 of pure Cu, as seen in Figure 15b. Another peak at 935.1 eV was assigned to Cu(OH)_2_, which suggested that elemental Cu was oxidized slightly at the low arcing rate (0.079%). This confirmed that the main wear mechanism was still mechanical wear at the low arcing rate and that the Cu was transferred from the disk by adhesive wear.

The accurate chemical composition of the spherical particles at a high arcing rate in Figure 13c was detected by XPS, as shown in Figure 15c. The core level Cu 2p3/2 at 933.6 eV and satellite peaks at a higher binding energy of 941.3 and 943.8 eV were in good accordance with CuO [28]. Arc heat produced by the intensive arc discharge generated instantaneous high temperatures above 3800 K (3527 °C) [29,30]. This exceeded the oxidation temperature (room temperature) and melting temperature (1083.4 °C) of Cu, which also reached the melting point of CuO (1026 °C). In this case, the melted Cu mixed with its oxidation product (CuO) splashed onto the pure carbon strip during the current-carrying sliding. At last, they solidified into spherical particles on the worn surface of the strip. Besides this, a bit of metal Cu was detected at 932.7 eV, which suggested that most of the transferred metal Cu was oxidized into CuO at the high arcing rate (16.141%). The main wear mechanism at the high arcing rate changed to arc erosion.

In summary, the dynamic contact between the contact wire and the pure carbon strip was reproduced on a pin-on-disk tribometer. The dynamic contact force caused continuous circulation of loading and unloading impacts which leaded to the unstable contact between the friction couples; consequently, arc discharges were produced. The dynamic contact force at different speeds can be described by a sine equation with different amplitude *B* and frequency *f*. The arcing rate of the strip ascended with the increase of *f* and *B*, and these influences were much more significant at high velocity. SEM and XPS analytical results demonstrated that mechanical wear was the dominant wear mechanism at a lower arcing rate while arc erosion had the leading effects at a higher arcing rate. These results show a deeper understanding of the damage to a pantograph/catenary system subjected to dynamic contact force, and will be beneficial for the safe operation of high-speed railway.

## 4. Conclusions

In this paper, arc discharges under dynamic contact force were studied at different speeds. The effects of amplitude *B* and frequency *f* on the arcing rate, current-carrying efficiency, and current-carrying stability were discussed. The surface damage mechanism under different arcing rates was analyzed using SEM and XPS. The conclusions are summarized as follows:

1. Arc discharges of the pure carbon strip were produced periodically in accordance with the period of the dynamic contact force. The arcing rate increased with the increase of velocity in the same test duration (40 s). What is more, the increase of *f* and *B* led to the rising of the arcing rate. The faster the train ran, the more influence *f* and *B* had.
2. Current-carrying qualities decreased with the increase of the arcing rate. A critical point of the arcing rate at around 2% was detected, lower than which the pure carbon strip maintained an excellent current-carrying capability. At a higher arcing rate, the current-carrying quality deteriorated abruptly.
3. Based on SEM and XPS, obvious scratches and scale-like debris were detected at low arcing rates. Metal Cu was transferred from the QCr0.5 disk to the pins by abrasive wear. At high arcing rates, cracks with a number of CuO spherical particles were captured. This means that the wear mechanism transferred from mechanical wear to arc erosion with the increase of the arcing rate.

## Figures and Tables

**Table 1:** Physical properties of the pure carbon strip.

Material	Density (kg·m^−3^)	Elasticity Modulus (GPa)	Electrical Resistivity (µΩ·m)	Bending Strength (MPa)	Hardness
Pure carbon	1.68	98.7	<40	>25	65 HS

**Table 2:** Chemical composition and mass fraction of QCr0.5 (wt %).

Composition	Pb	Zn	Fe	Sn	S	Si	Ni	Cr	Cu
QCr0.5	0.005	0.005	0.005	0.005	0.005	0.002	0.005	0.5	Bal.

**Table 3:** Amplitude–frequency characteristic of the dynamic contact force.

Velocity *v*/(km·h^−^^1^)	40	60	70	80	90	100
Amplitude *B* (N)	8	13	21	28	35	41
Frequency *f* (Hz)	0.169	0.254	0.296	0.338	0.380	0.426

**Table 4:** Dynamic contact force parameters in decoupling experiments.

Test	Velocity/( km·h^−^^1^)	Amplitude *B*/(N)	Frequency *f*/(Hz)
Test 1	40	8	0.169, 0.254, 0.338, 0.380, 0.426
	80	28	0.169, 0.254, 0.338, 0.380, 0.426
	100	41	0.169, 0.254, 0.338, 0.380, 0.426
Test 2	40	8, 13, 28, 35, 41	0.169
	80	8, 13, 28, 35, 41	0.338
	100	8, 13, 28, 35, 41	0.426
